# Structural Characterization and Emulsifying Properties of Highly Soluble Macadamia–Soybean Protein Composites Fabricated by Alkaline-Thermal Treatment

**DOI:** 10.3390/foods15030497

**Published:** 2026-02-01

**Authors:** Xiaohong He, Xixiang Shuai, Ming Zhang, Mingfeng Fang, Lei Zhao, Yunhui Cheng, Liqing Du

**Affiliations:** 1 South Subtropical Crops Research Institute, Chinese Academy of Tropical Agricultural Sciences, Key Laboratory of Tropical Fruit Biology, Ministry of Agriculture & Rural Affairs, Key Laboratory of Postharvest Physiology and Technology of Tropical Horticultural Products of Hainan Province, Zhanjiang 524091, China; hexiaohong@csust.edu.cn (X.H.); shuaixixiang1989@163.com (X.S.); zhangmingqau@163.com (M.Z.); ffmmff13@163.com (M.F.); 2 School of Food Science and Bioengineering, Changsha University of Science & Technology, Changsha 410114, China; 3 College of Food Science, South China Agricultural University, Guangzhou 510642, China; scauzl@scau.edu.cn

**Keywords:** protein interactions, alkaline-thermal treatment, solubility, structural analysis, emulsifying properties

## Abstract

The complementarity of plant proteins from various sources could achieve higher nutritional value to satisfy the requirement of replacing animal proteins. Therefore, it is very important to seek efficient and convenient approaches to fabricate highly soluble protein composites. In this study, macadamia protein–soybean protein (SP-MP′) composites were fabricated by alkaline-thermal treating at different ratios of 1:0.5, 1:1, and 1:2; then, the nitrogen solubility index, particle characteristics, and structure and emulsifying properties of SP-MP′ composites were investigated. The nitrogen solubility indexes of SP-MP′ composites were higher than 80%, and less small insoluble aggregates were observed by scanning electron microscopy. SP-MP′ composites exhibited high ζ-potential values, which were higher than −50 mV. Sodium dodecyl sulfate–polyacrylamide gel electrophoresis (SDS-PAGE) analysis found that both subunits of individually alkaline-thermal-treated macadamia protein (MP′) and soybean protein (SP′) were presented in SP-MP′ composites. The results of fluorescence, sulfhydryl group, and secondary structure illustrated that the SP interacted with MP to form SP-MP′ composites by the co-folding of proteins during neutralization. Compared to the individual proteins, SP-MP′ composites exhibited stronger emulsification ability and stability indexes (EAI and ESI) as the proportion of MP increased, and the EAI and ESI of SP-MP_1:2_′ were 21.53 m^2^/g and 146.7%, respectively. Meanwhile, emulsions prepared by SP-MP′ composites displayed more uniform oil droplet distributions. The findings suggested that highly soluble SP-MP′ composites with stronger emulsification abilities were successfully fabricated, which have great potential as ingredients to manufacture nutritional plant protein beverages.

## 1. Introduction

With the growth of population and the increasing attention of consumers to health and nutrition, the demand for plant proteins is growing due to its advantages of low cholesterol, low saturated fatty acids, and the environmentally friendly production process [1]. Exploring sustainable high-quality protein resources offered a viable solution to satisfy the rising demand of protein. The production of macadamia nuts in China is ranks among the top in the world, but the processing of macadamia nuts is still mainly based on primary processing, resulting in a low comprehensive utilization rate of the by-products from macadamia nuts. Macadamia protein (MP) is derived from oil-extracted macadamia meal, which contains approximately 30% protein [2,3]. Its amino acid profile is closely aligned with that proposed by the WHO/FAO, since it provides well-balanced essential amino acids for the human body [4]. Given its availability and nutritional value, macadamia protein was established as a high-quality plant protein source for food applications. Nevertheless, similar to other plant proteins, the low solubility of macadamia protein was an important factor to limit its application [5]. Macadamia protein is more soluble under acidic and alkaline conditions due to its high levels of asparagine/aspartic acid, glutamine/glutamic acid, and arginine [6]. Meanwhile, the molecular conformation and interactions of proteins are particularly sensitive to changes in pH of the aqueous medium [7]. In recent years, a prevalent strategy for protein modification involved partial unfolding of protein molecules into a molten globule state under highly alkaline conditions, followed by refolding at near-neutral pH [8,9].

However, protein aggregation often occurred during adjusting from alkalinity to neutral pH. When diverse proteins coexisted within a system, intermolecular non-covalent interactions would be formed between partially unfolded proteins, which might limit the exposure of hydrophobic groups to mitigate the tendency of aggregation [10]. Crucially, protein–protein interactions can enhance the functional properties of proteins, including solubility [11]. For instance, Wang et al. [12] fabricated a hydrophilic colloidal co-assemblies to improve water solubility by first hydrating rice and cod proteins at pH 12 and then neutralizing. Tilapia–soybean dual proteins were assembled by shifting pH from 12 to 7, and it was found that higher solubility, emulsifying, and foaming capacities were displayed [13]. Similarly, complexation between plant proteins via strong alkaline hydration and neutralization could simultaneously balance nutrition and enhance functionality. Complexing wheat gluten protein with soy protein increased the solubility of wheat gluten protein from 4.5% to 72.4% [14]. Complexation of rice protein with pea protein improved the levels of the limited amino acids (lysine in rice protein and sulfur-containing amino acids in pea protein), and the water solubility and digestibility of proteins were also improved [15]. Rao et al. [10] adopted alkaline-thermal treatment to prepare soluble oat–soybean and oat–pea protein composites, which was conducted by incubating a protein mixture at pH 12 and 55 °C for 1.5 h, then cooling it to ambient temperature and neutralizing it to pH 7. Despite this potential, there is currently no research on the fabrication of protein composites containing macadamia protein and other plant proteins via alkaline-thermal treatment. Soy protein (SP), a widely utilized legume protein in the food industry, is limited in essential sulfur-containing amino acids [16]. Macadamia–soy protein composites prepared by the alkaline-thermal method would compensate for the lack of essential sulfur-containing amino acids in soy protein. Simultaneously, in order to better apply protein composites, their structural characteristics and functional attributes, such as solubility and emulsifying properties, need to be further explored. Successfully constructing a highly soluble macadamia–soy protein composites would significantly broaden the application potential of macadamia protein in foods.

Therefore, this study aimed to fabricate macadamia protein–soy protein composites by subjecting the mixture of macadamia protein and soy protein to hydrate at pH 11 and 55 °C to promote protein unfolding, followed by neutralizing to pH 7 to facilitate refolding. The nitrogen solubility indexes of protein composites were determined using the Kjeldahl method. Particle characteristics were analyzed via scanning electron microscopy (SEM), particle size, and zeta potential measurements. The molecular binding, refolding, and complexation of SP and MP were studied by SDS-PAGE, fluorescence spectroscopy, and circular dichroism (CD). Finally, the emulsifying ability (EAI) and emulsifying stability index (ESI) of macadamia–soy protein (SP-MP′) composites, alongside the particle size and optical microscopic images of emulsions prepared using SP-MP′ composites, were used to evaluate the emulsifying properties. The novel nutritious protein composites obtained in this study may be expected to be applied as ingredients for the development of high-value plant-based protein beverages.

## 2. Materials and Methods

### 2.1. Material

Macadamia nuts were provided by the National Macadamia Germplasm Repository (Zhanjiang, Guangdong Province, China; Longitude: 110°27′56.1″ E, Latitude: 21°16′32.5″ N). Commercial soybean protein isolates were obtained from Yuanye Bio-Technology Inc. (Shanghai, China), with a composition of 83.9% protein, 7.1% moisture, 3.9% ash, and 0.2% fat. 1-Anilino-8-naphthalene-sulfonate (ANS), glycine and tris base were obtained from Sigma-Aldrich (Shanghai, China). 5,5-Dithiobis-(2-nitrobenzoic acid) (DTNB) and Coomassie brilliant blue G-250 were purchased from Aladdin Reagent Company (Shanghai, China). The protein marker (ColorMixed Protein Marker, ranging from 11~180 kDa) and urea were purchased from Solarbio Science and Technology (Beijing, China). Hydrochloric acid (HCl) and sodium hydroxide (NaOH) were sourced from Xilong Scientific Co., Ltd. (Shantou, Guangdong Province, China). Food-grade soybean oil was supplied by Yihai Kerry Golden Dragon Fish Food Group Co., Ltd. (Shanghai, China). All other chemicals used were of analytical grade. Deionized water was used for preparing all aqueous solutions.

### 2.2. Extraction of Macadamia Protein (MP) Isolates

Macadamia protein isolates were extracted following a previously described method by Zhong et al. [4] with slight modifications. Briefly, macadamia kernels were finely ground. To remove lipids, the kernel slurry was mixed with petroleum ether (1:10, *w*/*v*) at room temperature (25 °C) under constant stirring for 24 h. The dispersion was then filtered through a Buchner funnel (Chengdu Dianrui Experimental Instrument Co., Ltd., Chengdu, Sichuan Province, China) to collect the defatted macadamia meal. The defatting procedure was repeated in triplicate. Residual solvent was evaporated from the meal by air-drying in a fume hood. Subsequently, the defatted meal was dispersed in deionized water (1:20, *w*/*v*) at room temperature. The pH of suspension was adjusted to 11.0 with 3 mol/L NaOH. After continuous stirring for 2 h, the mixture was centrifuged at 8000× *g* for 20 min using a large-capacity centrifuge (LXJ-IIB, Youyi Instrument Co., Ltd., Shanghai, China). The supernatant was collected, and proteins were precipitated at the isoelectric point by adjusting the pH of the supernatant to 4.5 with a certain concentration of HCl and NaOH solution [2]. The precipitate was recovered by centrifugation under the same conditions as described above. The protein pellet was washed three times using distilled water with pH of 4.5 to remove the salts and purify the protein, and further water washing was carried out twice to return the protein to neutrality. Finally, the purified protein was freeze-dried and stored in a desiccator until analysis. The purity of the protein was determined using the Kjeldahl method with an automatic Kieldahl apparatus (K9840, Haineng Instruments Co., Ltd., Qingdao, China).

### 2.3. Preparation of Macadamia–Soybean Protein (SP-MP′) Composites

SP-MP′ composites were prepared via alkaline-thermal treatment according to the method described by Rao et al. [10]. Briefly, soybean protein was dispersed in deionized water to maintain a constant concentration of 1% (*w*/*v*), and the mass ratios of the SP/MP composites were 1:0.5, 1:1, and 1:2. The mixtures of SP and MP were first adjusted to pH 11 using 1 mol/L NaOH, followed by incubating at 50 °C with continuous stirring for 1 h. Subsequently, pH of the mixtures was adjusted back to 7 with 0.1 mol/L HCl. The SP-MP′ composites obtained at SP/MP mass ratios of 1:0.5, 1:1, and 1:2 were denoted as SP-MP_1:0.5_′, SP-MP_1:1_′, and SP-MP_1:2_′, respectively. In addition, 1% (*w*/*v*) of SP and MP were individually alkaline-thermal treated as described above, and named as SP′ and MP′. Untreated native soybean protein was designated as SP_native_, which served as the control. A portion of samples was centrifuged at 8000× *g* for 30 min to collect the supernatant. Then, the supernatant was dialyzed overnight in deionized water at 4 °C using a dialysis bag (with a cut-off molecular weight of 3500 Da) to remove salt, which was used to analyze particle characteristics and structure, such as intrinsic fluorescence, surface hydrophobicity, circular dichroism, sulfhydryl and disulfide bond content, and sodium dodecyl sulfate–polyacrylamide gel electrophoresis (SDS-PAGE). In addition, the remaining uncentrifuged samples were dialyzed overnight in deionized water at 4 °C to remove salt and were used to determine the microstructure and evaluate the emulsifying properties.

### 2.4. Determination of Nitrogen Solubility Index

The nitrogen solubility index of the proteins was assessed as described in a previous report [17]. After centrifugation (8000× *g*, 20 min), the nitrogen contents of the resulting supernatants were quantified by the Kjeldahl nitrogen method, using an automatic Kjeldahl apparatus (K9840, Haineng Instruments Co., Ltd., Qingdao, China). The nitrogen solubility index was calculated as the ratio of the soluble nitrogen content in the supernatant to the total nitrogen content in the whole dispersion.

### 2.5. Scanning Electron Microscopy (SEM)

The morphology of the SP-MP′ composites was examined according to the methodology described by Li et al. [18]. Samples were prepared by depositing the protein suspension onto monocrystalline silicon wafers and allowing them to dry. The dried silicon wafers were then affixed to conductive adhesive tape, which was mounted on circular specimen stub. Subsequently, samples were sputter-coated with a thin film of gold, and observed at ×500 magnifications and an accelerating voltage of 5.0 kV using a scanning electron microscope (SEM, Quanta-200, FEI Company, Eindhoven, The Netherlands).

### 2.6. Particle Size and Zeta Potential Analysis

The average particle size and ζ-potential of the SP-MP′ composites were measured using a dynamic light-scattering/electrophoresis instrument (Zetasizer Nano-ZS, Malvern Ltd., Malvern, Worcestershire, UK) by referencing the method of Gao, Rao, and Chen [19]. Prior to measurement, the samples were diluted to a uniform concentration (0.1 mg/mL) using deionized water. Measurements were performed at ambient temperature. The refractive index values of the protein and water were 1.45 and 1.33, respectively.

### 2.7. Characterization of Protein Structure

#### 2.7.1. Sodium Dodecyl Sulfate–Polyacrylamide Gel Electrophoresis (SDS-PAGE)

The SDS-PAGE profiles of the SP-MP′ composites were analyzed as described by Yang et al. [20], with minor modifications. Briefly, the dialyzed SP-MP′ composite supernatant was diluted to 2.0 mg/mL according to the calculated soluble protein content in Section 2.4. A total of 30 μL of SP-MP′ composite solution was mixed with 10 μL of 4× loading buffer containing 1,4-Dithiothreitol. The mixture was heated at 100 °C for 7 min in a water bath to denature the protein, followed by centrifugation at 12,000× *g* for 5 min. After cooling to room temperature, 10 μL of supernatant was carefully loaded onto an SDS-PAGE gel, which comprised a 12% separating gel and a 5% stacking gel. A sample containing 20 μg protein was loaded into each lane. Simultaneously, the protein marker was loaded as a molecular weight standard. Electrophoresis was initially performed at 80 V for 15 min to facilitate protein migration through the stacking gel, and the voltage was subsequently increased to 120 V for 40 min to achieve protein separation in the separating gel. After electrophoresis, gels were stained with 0.25% (*w*/*v*) Coomassie brilliant blue R-250 in a solution containing 50% (*v*/*v*) methanol and 10% (*v*/*v*) acetic acid for 1 h at room temperature. Excess dye was removed by destaining in a solution containing 10% (*v*/*v*) acetic acid to visualize the protein bands.

#### 2.7.2. Intrinsic Fluorescence Analysis

The intrinsic fluorescence spectra of SP-MP′ composites were documented using a fluorescence spectrophotometer (Hitachi F-7000, Hitachi, Ltd., Tokyo, Japan) in emission scan mode. The samples were diluted to 0.2 mg/mL with deionized water for measurements. Fluorescence spectra were collected over a wavelength range of 290~500 nm at an excitation wavelength of 280 nm. The emission slit widths were 5.0 nm, and the scan speed was 1000 nm/min. The detector voltage and response time were 700 V and 0.5 s, respectively.

#### 2.7.3. Determination of Surface Hydrophobicity (H_0_)

Surface hydrophobicity (H_0_) of the SP-MP′ composites was determined using a fluorescence spectrophotometer (Hitachi F-7000, Hitachi, Ltd., Tokyo, Japan), according to the method of He et al. [21] with minor modifications. Protein solutions were diluted to different concentrations ranging from 0.002% to 0.010% (*w*/*v*). Subsequently, an aliquot (10 μL) of freshly prepared 1-anilino-8-naphthalene-sulfonate solution (8 mM) was added into 5 mL of the dilution, then mixed and kept in dark for 15 min at room temperature. The extrinsic ANS fluorescence intensity was measured at an excitation wavelength of 390 nm and an emission wavelength from 400 to 800 nm. The excitation and emission slits were both 2.5 nm and the scan speed was 1200 nm/min. H_0_ was calculated as the initial slope of the linear regression plot of maximal fluorescence intensity versus protein concentration.

#### 2.7.4. Circular Dichroism (CD)

CD spectra of the SP-MP′ composites (1.0 mg/mL) were recorded using a circular dichroism spectrometer (MOS-450, Bio-Logic SAS, Grenoble, France) over the wavelength range of 190~250 nm at room temperature. The size of cuvette was 5.5 × 1.2 cm, and the optical distance was 1 mm. The extract secondary structure content was deconvoluted and calculated according to DICHROWEB CONTIN (2021) online.

#### 2.7.5. Determination of Sulfhydryl Groups (SH) and Disulfide Bond (SS)

The content of sulfhydryl groups and disulfide bonds of SP-MP′ composites was determined according to the method described by Xiao et al. [22]. SP-MP′ composite supernatant was diluted to 1 mg/mL using Tri-Gly buffer (0.086 mol/L Tris, 0.09 mol/L glycine, 0.004 mol/L ethylenediamine tetraacetic acid, and 8 mol/L urea). Subsequently, 250 μL of Ellman’s reagent (4 mg/mL 5,5-Dithiobis-(2-nitrobenzoic acid) (DTNB) in Tris-glycine buffer) was added to 5 mL of the diluted sample. The mixture was incubated at 37 °C for 1 h, and absorbance at 412 nm was measured using a UV spectrophotometer with blank correction. A molar extinction coefficient was used to calculate the content of SH. The free sulfhydryl content was then calculated using Equation (1):

(1) $$\mathrm{SH}\;\left(\mu mol/g\right)=\frac{73.53\times A\times D}{C}$$

Here, 73.53 is the molar absorbance coefficient of DTNB, A is the absorbance of the sample at 412 nm, D is the dilution factor, and C is the protein concentration (1 mg/mL).

To determine total sulfhydryl content, 5 mL of the diluted SP-MP′ composite supernatant was mixed with 0.1 mL β-mercaptoethanol and incubated at 37 °C for 1 h. The mixture was then precipitated with 15 mL of 12% trichloroacetic acid (TCA) and centrifuged at 4800× *g* for 15 min. After discarding the supernatant, the precipitate was collected, and the procedure of precipitation was repeated twice to obtain the pellet. Subsequently, the pellet was redissolved in 5 mL Tris-Gly buffer, and 2 mL of solution was combined with 2 mL Tris-glycine buffer and 50 μL Ellman′s reagent, followed by incubation at 37 °C for 1 h. Absorbance was measured at 412 nm. Total sulfhydryl content was then calculated using Equation (1), while the content of disulfide bonds was derived from Equation (2) as half of the difference between the total and free sulfhydryl contents.

(2) $$S\text{-}S\;\left(\mu mol/g\right)=\frac{{\mathrm{SH}}_{\mathrm{total}}-{\mathrm{SH}}_{\mathrm{free}}}{2}$$

Here, SH_total_ is the total sulfhydryl content and SH_free_ is the free sulfhydryl content.

### 2.8. Emulsifying Properties of SP-MP′ Composites

#### 2.8.1. Emulsifying Activity Index (EAI) and Emulsion Stability Index (ESI)

The ability of SP-MP′ composites to form and stabilize emulsions was evaluated following the method of Tan et al. [23] with minor modifications. Briefly, 12 mL of SP-MP′ composite (10 mg/mL, pH 7.0) was mixed with 3 mL of soybean oil, and homogenized for 1 min at 10,000 rpm using a high-speed disperser (T18BS25, IKA, Staufen, Germany). Subsequently, 50 μL aliquots of the freshly formed emulsion were withdrawn from a constant location within the container and mixed with 5 mL of 0.1% (*w*/*v*) sodium dodecyl sulfate (SDS). The absorbance of the diluted emulsions was measured using a UV–visible spectrophotometer (TU-1901, Persee Instrument Co., Ltd., Beijing, China) with a wavelength of 500 nm, immediately (A_0_) and after 30 min (A_30_). The EAI and ESI values were calculated according to Equations (3) and (4), respectively.

(3) $$\mathrm{EAI}=\frac{2\times 2.303\times A_{0}\times D}{C\times \left(1-\varphi \right)\times L\times 10000}$$

(4) $$\mathrm{ESI}=\frac{A_{0}\times \Delta t}{A_{0}-A_{30}}$$

Here, D is the dilution factor, C is the protein concentration (g/mL), L is the optical pathlength (1 cm), φ is the volume fraction of oil (L/L), A_0_ is the absorbance value at 0 min, A_30_ is the absorbance value after 30 min, and Δt is the time interval (30 min).

#### 2.8.2. Particle Size Distribution of Emulsions Prepared by SP-MP′ Composites

The particle size distribution of emulsions was characterized using a laser diffraction particle size analyzer (Mastersizer 3000, Malvern Instruments Ltd., Malvern, UK) as described by Zeng et al. [24]. In brief, emulsions were introduced dropwise into a measurement cell filled with deionized water until an appropriate obscuration was reached. Refractive indices were set at 1.465 for soybean oil and 1.33 for the aqueous phase. Prior to measurement, samples were dispersed at 2000 rpm for 20 s. The droplet size distribution and average particle diameters (expressed as D_[3,2]_) were recorded.

#### 2.8.3. Micromorphology of Emulsions Prepared by SP-MP′ Composites

The emulsions were prepared using a two-stage method. First, the SP-MP′ composite prepared in Section 2.3 was diluted to 10 mg/mL. A mixture of soybean oil (5%, *v*/*v*) and SP-MP′ composite (95%, *v*/*v*) was homogenized using an Ultraturrax T18 homogenizer (IKA, Staufen, Germany) at 12,000 rpm for 2 min to form a coarse emulsion. Subsequently, the coarse emulsion was further homogenized using a high-pressure microfluidizer at 80 MPa for three cycles to obtain the final SP-MP′ composite-stabilized emulsion. Emulsions prepared with SP_native_, SP′, MP′, SP-MP_1:0.5_′, SP-MP_1:1_′, and SP-MP_1:2_′ were designated as E_SPnative_, E_SP′_, E_MP′_, E_SP-MP1:0.5′_, E_SP-MP1:1′_, and E_SP-MP1:2′_, respectively. A droplet of each emulsion was deposited on a glass microscope slide and covered with a coverslip. The micromorphology of emulsions was observed under a microscope (Olympus CX31, Tokyo, Japan) equipped with a DC 3000 camera at a magnification of 10 × 10.

### 2.9. Data Analysis

All experiments were carried out in triplicate using three samples, and then the mean and standard deviation were calculated by statistical analysis software (SPASS 25.0, Chicago, IL, USA). Significant differences between sample means (*p* < 0.05) were established according to Duncan′s test using one-way analysis of variance (ANOVA).

## 3. Results and Discussion

### 3.1. Nitrogen Solubility Index

Plant proteins typically exhibited incomplete dissolution or low solubility, which led to suboptimal techno-functional properties, making it challenging for their application in food formulations. Therefore, enhancing the solubility of proteins would improve their functional properties, such as emulsification and foaming [25]. Consequently, determining the nitrogen solubility index was essential prior to application, particularly for the co-assembled plant protein composites. Table 1 presents the nitrogen solubility index of the SP-MP′ composites. SP_native_ showed a solubility index of 28.6%, and that of native macadamia protein (MP_native_) was 11.2%. After alkaline-thermal treatment, the solubility indexes of SP′ and MP′ were significantly increased to 90.1% and 94.0%, respectively. The increase in solubility was primarily attributed to alkaline-induced structural modifications of protein molecules. Additionally, the conversion of side-chain amide groups into the charged acids increased protein hydrophilicity, which was another factor affecting solubility after alkaline-thermal treatment [18]. In regard to SP-MP_1:0.5_′, SP-MP_1:1_′, and SP-MP_1:2_′, the nitrogen solubility indexes were 81.1%, 82.6%, and 84.9%, respectively. The nitrogen solubility indexes of SP-MP′ composites were higher than 80%, which were better than the results reported by He et al. [14], who improved the solubility of wheat gluten protein to 72.4% after a pH-driven interaction with soy protein isolates. The solubility of the SP-MP_1:2_′ composite was lower than individually alkaline-thermal-treated MP and SP (namely MP′ and SP′). This may be due to the interactions between macadamia protein and soybean protein, which affected the number and orientation of hydrophilic groups.

### 3.2. Morphology

**Figure 1:**
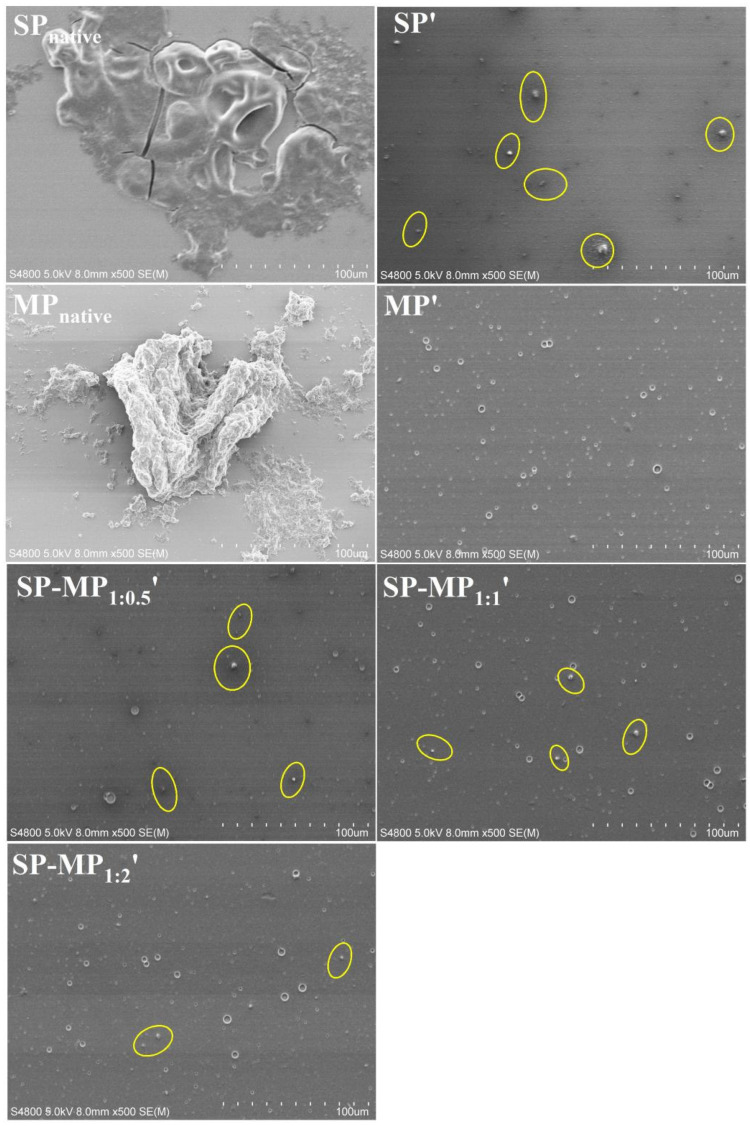
Scanning electron microscopy images of SP-MP′ composites. Magnification was 500×. SP_native_ indicates native soybean protein; SP′ and MP′, respectively, represent soybean protein and macadamia protein after alkaline-thermal treatment; SP-MP_1:0.5_′, SP-MP_1:1_′, and SP-MP_1:2_′ represent SP-MP′ composites obtained by alkaline-thermal treatment at SP/MP mass ratios of 1:0.5, 1:1, and 1:2, respectively.

Scanning electron microscopy (SEM) was employed to investigate microstructural changes in protein particles induced by alkaline-thermal treatment [10]. As depicted in Figure 1, it was observed that SP_native_ contained large irregularly shaped aggregates, which might be the insoluble protein bodies. After alkaline-thermal treatment, the large irregular-shaped aggregates of soybean protein disappeared, and they were transformed into smaller granular particles (as yellow circles in Figure 1). MP_native_ exhibited large insoluble block morphology. Concurrently, MP′ displayed fewer particulate structures, and smaller dot-like features were observed. This indicated that there were very few undissolved aggregates in MP′, confirming the results of the nitrogen solubility index that was close to complete dissolution. Given that macadamia protein after alkaline-thermal treatment was almost dissolved, native macadamia protein was not used as a control in subsequent experiments. Observing the microscopic images of SP-MP_1:0.5_′, SP-MP_1:1_′, and SP-MP_1:2_′, it was found that the SP-MP′ composites revealed a progressive reduction in small particle aggregates with the increase in the MP ratio. Notably, SP-MP_1:2_′ showed minimal granular particulate substances. This trend aligned with the result of the nitrogen solubility index, suggesting that a higher proportion of MP enhanced the formation of water-dispersible proteins in SP-MP′ composites.

### 3.3. Particle Size and ζ-Potential

**Figure 2:**
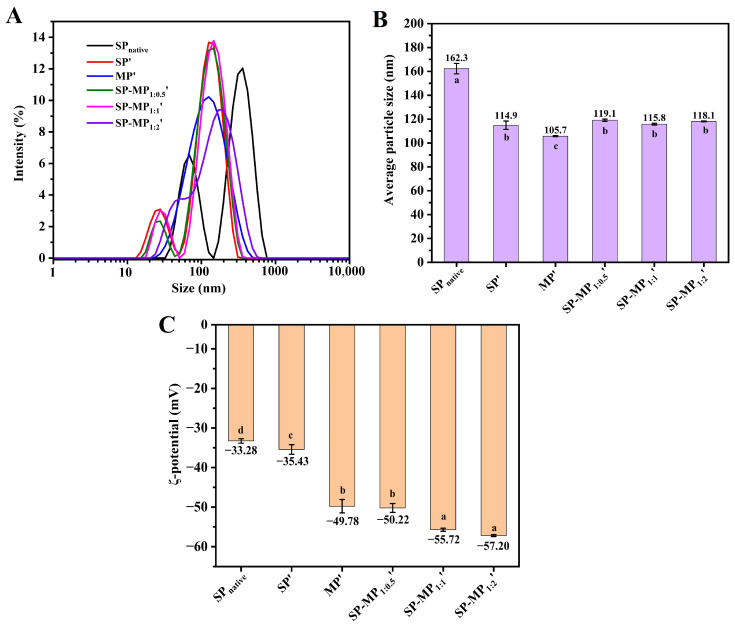
Particle size distribution (**A**), average particle size (**B**), and ζ-potential (**C**) of SP-MP′ composites. SP_native_ indicates native soybean protein; SP′ and MP′, respectively, represent soybean protein and macadamia protein after alkaline-thermal treatment; SP-MP_1:0.5_′, SP-MP_1:1_′, and SP-MP_1:2_′ represent SP-MP′ composites obtained by alkaline-thermal treatment at SP/MP mass ratios of 1:0.5, 1:1, and 1:2, respectively. The lowercase letters in each subfigure indicate significant differences (*p* < 0.05).

The particle size and ζ-potential of the colloidal particles in a protein suspension have an influence on their water-dispersibility, and they could be characterized by dynamic light-scattering (DLS) measurements. As illustrated in Figure 2A, SP_native_ exhibited a bimodal particle size distribution, whereas that of SP′ showed a leftward shift, indicating that alkaline-thermal treatment reduced the particle size of soybean protein. The average particle size of SP_native_ was 162.3 nm, which decreased significantly to 114.9 nm for SP′ (Figure 2B). The particle size distribution of MP′ displayed a unimodal pattern with an average particle size of 105.7 nm. Notably, SP-MP′ composites maintained a bimodal distribution, and the predominant peak position was aligned closely with that of SP′. The average particle sizes of SP-MP_1:0.5_′, SP-MP_1:1_′, and SP-MP_1:2_′ were 119.1, 115.8, and 118.1 nm, respectively, which were slightly lower than MP′ but comparable to SP′, suggesting that alkaline-thermal treatment could yield soluble protein composites with smaller particle sizes.

The surface charge of protein particles was characterized by measuring the changes in ζ-potential, as shown in Figure 2C. The ζ-potential value of SP_native_ was −33.28 mV, and the absolute ζ-potential value of the protein increased after alkaline-thermal treatment, which was similar to the change in interfacial potential under slightly alkaline-thermal treatment (pH < 9.0) in the reports of Zhang et al. [26]. The ζ-potential values of SP′, MP′, SP-MP_1:0.5_′, SP-MP_1:1_′, and SP-MP_1:2_′ were −35.43, −49.78, −50.22, −55.72, and −57.20 mV, respectively. SP-MP′ composites exhibited higher absolute ζ-potentials than individual SP′ and MP′, and they gradually increased with the increase in MP ratio. This enhancement was likely attributed to conformational changes and chemical modifications of the charged groups induced by alkaline-thermal treatment, which exposed additional charged amino acids [18]. The elevated ζ-potentials strengthened interparticle electrostatic repulsions, thereby enhancing the ability of protein to resist aggregation.

### 3.4. SDS-PAGE

**Figure 3:**
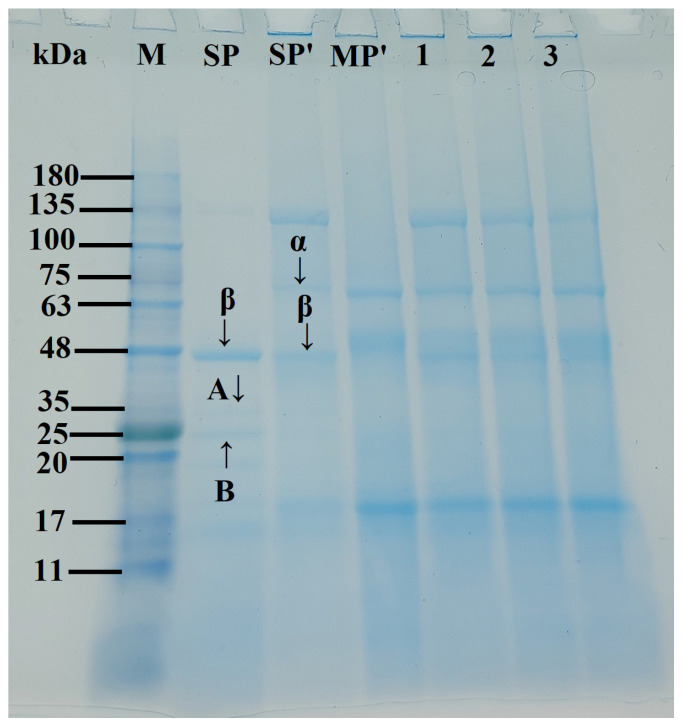
SDS-PAGE profiles of SP-MP′ composites; Lane M represents markers; Lane SP_native_ indicates native soybean protein; Lane SP′ and MP′, respectively, represent soybean protein and macadamia protein after alkaline-thermal treatment; Lane 1~3 are SP-MP_1:0.5_′, SP-MP_1:1_′, and SP-MP_1:2_′, which represent SP-MP′ composites obtained by alkaline-thermal treatment at SP/MP mass ratios of 1:0.5, 1:1, and 1:2, respectively. α and β represent the two subunits of β-conglycinin (7S); A and B represent the acidic and basic subunits of glycinin (11S), respectively.

Figure 3 presents the SDS-PAGE electrophoretic profiles of SP-MP′ composites. In SP and SP′ samples, β-conglycinin (7S) subunits (α and β) and glycinin (11S) subunits (acidic A and basic B) were observed, which was consistent with the reports of Li et al. [27] and Yang et al. [28]. Notably, alkaline-thermal treatment induced an additional prominent band at 100~135 kDa for SP′. MP′ exhibited characteristic bands at ~18, 52, and 62 kDa. For SP-MP′ composites, major bands from both SP′ (α, β, and 100~135 kDa) and MP′ (~18, ~52, ~62 kDa) were presented in the electrophoresis pattern, indicating that molecular interactions between soybean protein and macadamia protein occurred [29]. As the ratio of MP increased, the intensity band at ~18 kDa in SP-MP′ composites progressively intensified, while the band at 100~135 kDa faded. These electrophoretic features collectively demonstrated the successful fabrication of SP-MP′ protein composites.

### 3.5. Intrinsic Fluorescence Spectroscopy

**Figure 4:**
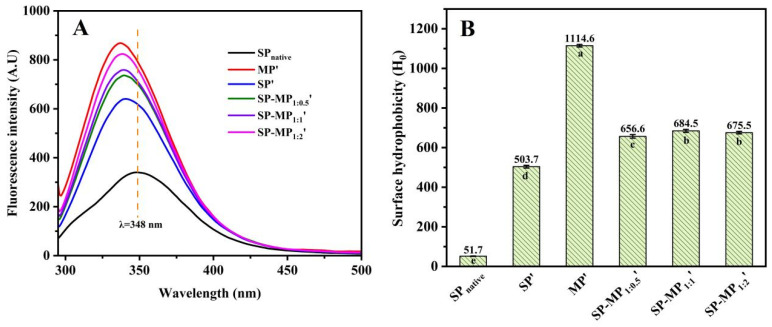
Intrinsic fluorescence spectrum (**A**) and surface hydrophobicity (**B**) of SP-MP′ composites. SP_native_ indicates native soybean protein; SP′ and MP′, respectively, represent soybean protein and macadamia protein after alkaline-thermal treatment; SP-MP_1:0.5_′, SP-MP_1:1_′, and SP-MP_1:2_′ represent SP-MP′ composites obtained by alkaline-thermal treatment at SP/MP mass ratios of 1:0.5, 1:1, and 1:2, respectively. The lowercase letters in each subfigure indicate significant differences (*p* < 0.05).

Usually, hydrophilic groups of natural proteins are oriented externally, while hydrophobic groups are isolated internally, encapsulating most chromophores within the molecular structure [30]. Intrinsic fluorescence emitted from aromatic amino acid residues, such as tryptophan, tyrosine, and phenylalanine, serves as a sensitive indicator of tertiary structural alterations, as the shifts in the maximum emission wavelength and intensity reflect the changes in the local environment of these residues. Figure 4A presents the intrinsic fluorescence spectra of SP-MP′ composites. SP_native_ exhibited a maximum fluorescence intensity (F_max_) at λ_max_ = 348 nm. SP′ displayed a blue shift in λ_max_ to 341 nm, which was accompanied by a significant increase in F_max_ to 640.3. This shift and intensity enhancement were attributed to the increase in exposure of chromophore groups from intramolecular hydrophobic domains to the polar aqueous environment [31], since alkaline-thermal treatment induced protein conformational changes or dissociation of aggregates. MP′ showed an F_max_ of 868.5 at λ_max_ = 337 nm. All SP-MP′ composites exhibited λ_max_ values near 340 nm, which were between the values of their individual proteins after alkaline-thermal treatment. A similar phenomenon was found in the fluorescence spectroscopy of oat protein–soy protein composites prepared by alkaline-thermal treatment [10]. The F_max_ values of SP-MP_1:0.5_′, SP-MP_1:1_′, and SP-MP_1:2_′ were 736.2, 758.8, and 823.9 nm, respectively. The F_max_ values of SP-MP′ composites were intermediate between those of SP′ and MP′, which might be due to the interactions between soy proteins and macadamia proteins altering the microenvironment of non-polar aromatic amino acids.

### 3.6. Surface Hydrophobicity (H_0_)

The ANS fluorescence method could be used to obtain surface hydrophobicity information of SP-MP′ composites, which is mainly based on the binding of hydrophobic probes to non-polar groups on the protein surface [32]. As shown in Figure 4B, SP_native_ exhibited the lowest surface hydrophobicity (51.7). During alkaline-thermal treatment, proteins undergo significant unfolding and refolding, and their surface hydrophobicity is substantially altered. Consequently, the surface hydrophobicity of SP′ was nearly 10-fold higher (503.7) than SP_native_. As analyzed by Xu et al. [31], deprotonation of proteins caused by extreme alkaline pH disrupted the hydrophobic interactions within and between soybean protein molecules, thereby exposing the hydrophobic groups to the surface. The H_0_ of MP′ was 1114.6, and the surface hydrophobicity values of SP-MP_1:0.5_′, SP-MP_1:1_′, and SP-MP_1:2_′ were 656.6, 684.5, and 675.5, respectively. According to the surface hydrophobicity values of SP′ and MP′, the theoretical calculation values (sum of the surface hydrophobicity values of MP′ and SP′ with corresponding proportion) of SP-MP_1:0.5_′, SP-MP_1:1_′, and SP-MP_1:2_′ were 707.1, 809.5, and 911.2, respectively. Therefore, it was found that the H_0_ of SP-MP′ composites was actually lower than the theoretical calculation value of SP′ and MP′ mixtures. The result indicated that the formation of protein composites during alkaline-thermal treatment had an impact on the molecular structure of proteins. The lower surface hydrophobicity values suggested that the hydrophobic domains of protein were sheltered, providing further evidence of molecular associations and interactions between the proteins. Additionally, the H_0_ of SP-MP′ composites was higher than SP′ but lower than MP′. Sun et al. [30], who reported that a soy protein/potato protein complex exhibited a lower H_0_ compared with both soybean protein and pea protein after pH-shifting treatment, explained that the formation of soluble aggregates was involved in hydrophobic interactions between soy protein and potato protein to induce an increase in hydrophilicity. The discrepancy of altering H_0_ may be related to the type of protein during the formation of the composites of soybean protein and other plant proteins.

### 3.7. Circular Dichroism

Circular dichroism spectroscopy was employed to characterize the secondary structures of SP-MP′ composites. Table 1 summarized the calculated contents of α-helix, β-sheet, β-turn, and random coil derived from the CD spectral analysis. The CD spectrum of SP_native_ exhibited a prominent negative peak near 215 nm. After alkaline-thermal treatment, the peak shifted to around 221 nm, indicating a change in the secondary structure of soy protein. Meanwhile, a decrease in its α-helix and an increase in β-sheet were found. The wavelength at the maximum negative peak in the circular dichroism spectrum of MP′ was similar to that of SP′, but MP′ had a higher α-helix and lower β-sheet and β-turn compared to SP′. The maximum negative peak wavelength of the SP-MP′ composites was about 220 nm. The contents of α-helix and β-sheet of SP-MP_1:0.5_′, SP-MP_1:1_′, and SP-MP_1:2_′ were intermediate between those of SP′ and MP′. As the proportion of MP increased, the α-helix of SP-MP′ composites progressively reduced, demonstrating that the complexation driven by alkaline-thermal treatment contributed to the formation of SP-MP′ composites with less ordered secondary structure.

### 3.8. Sulfhydryl Group and Disulfide Bond

**Figure 5:**
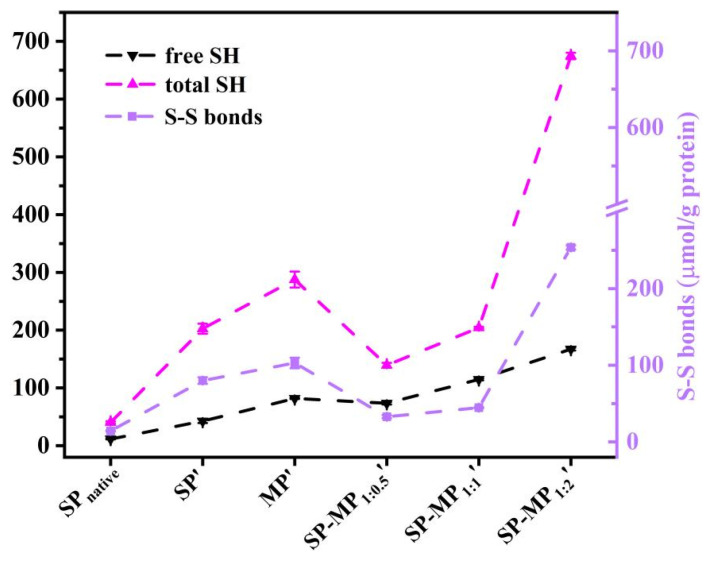
The free sulfhydryl group (SH_free_), total sulfhydryl group (SH_total_), and disulfide bond (S-S bond) of SP-MP′ composites. SP_native_ indicates native soybean protein; SP′ and MP′, respectively, represent soybean protein and macadamia protein after alkaline-thermal treatment; SP-MP_1:0.5_′, SP-MP_1:1_′, and SP-MP_1:2_′ represent SP-MP′ composites obtained by alkaline-thermal treatment at SP/MP mass ratios of 1:0.5, 1:1, and 1:2, respectively.

As shown in Figure 5, total sulfhydryl (SH_total_), free sulfhydryl (SH_free_), and disulfide bond (S-S bond) contents were determined in the supernatant of SP-MP′ composites. The total and free SH groups in SP′ were higher than those in SP_native_, indicating that strong alkaline conditions increased the number of sulfhydryl group. The increase in the free SH content of soybean protein was similar to the change induced by alkaline pH-shifting treatment [33]. This phenomenon might be attributed to the disruption of large protein aggregates and changes in protein conformation, leading to greater accessibility of sulfhydryl groups on protein surfaces [28]. MP′ demonstrated substantially higher SH_total_ and SH_free_ contents (287.7 and 81.8 μmol/g, respectively) than SP′, which may be attributed to the higher methionine levels in macadamia protein. In SP-MP′ composites, SH_free_ and S-S bond contents significantly varied with the increase in MP ratio, and SP-MP_1:2_′ showed the most pronounced elevation in S-S bond content. Notably, SP-MP_1:0.5_′ and SP-MP_1:1_′ exhibited lower disulfide bond contents than SP′ and MP′, whereas SP-MP_1:2_′ composites exceeded the disulfide bond content of both SP′ and MP′. Interactions between the soy protein and potato protein promoted the formation of disulfide bonds to reduce the SH_free_ content during pH-shifting treatment [29]. The degree of change in the SH_free_ and S-S bond contents of SP-MP′ composites during fabrication may have a relationship with the ratio of MP and methionine levels. It might be caused by the dynamic changes in breaking disulfide bonds and generating new disulfide bonds after subjecting to a condition of strong alkalinity.

### 3.9. Emulsifying Properties

#### 3.9.1. Emulsifying Activity and Emulsion Stability

**Figure 6:**
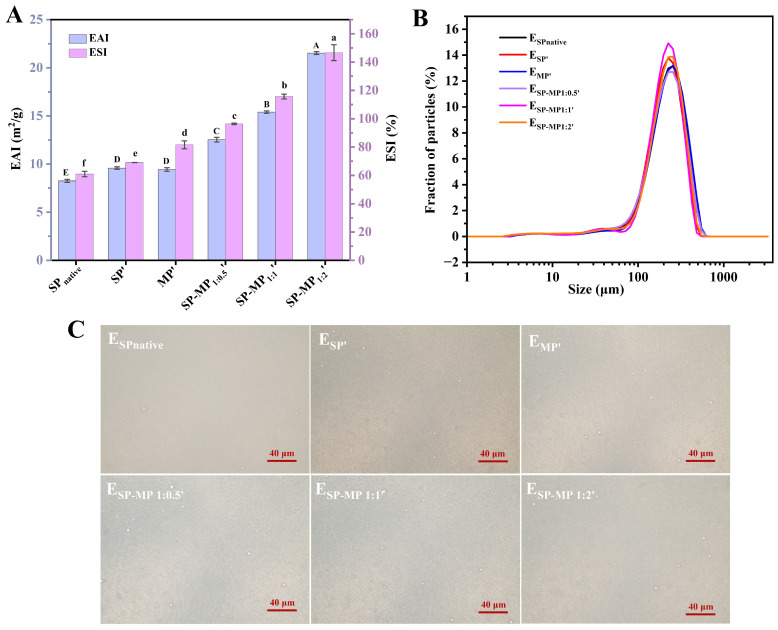
Emulsifying activity index (EAI) and emulsion stability index (ESI) of SP-MP′ composites (**A**); particle size distribution of emulsions stabilized by SP-MP′ composites (**B**); and optical microscopy images of emulsions stabilized by SP-MP′ composites (**C**). SP_native_ indicates native soybean protein; SP′ and MP′, respectively, represent soybean protein and macadamia protein after alkaline-thermal treatment; SP-MP_1:0.5_′, SP-MP_1:1_′, and SP-MP_1:2_′ represent SP-MP′ composites obtained by alkaline-thermal treatment at SP/MP mass ratios of 1:0.5, 1:1, and 1:2, respectively. E_SPnative_, E_SP′_, E_MP′_, E_SP-MP1:0.5′_, E_SP-MP1:1′_, and E_SP-MP1:2′_ represent the emulsions prepared with SP_native_, SP′, MP′, SP-MP_1:0.5_′, SP-MP_1:1_′, and SP-MP_1:2_′, respectively. The capital and lowercase letters in the subfigure A respectively indicate significant differences of emulsifying activity index and emulsion stability index (*p* < 0.05).

The emulsion activity index (EAI) quantifies protein adsorption capacity at the oil–water interface during homogenization, while the emulsion stability index (ESI) reflects the ability to stabilize oil droplets after homogenization. Figure 6A shows the emulsifying ability and stability of SP-MP′ composites. Compared to SP_native_, alkaline-thermal treatment marginally increased the EAI of soybean protein, and the EAI value of MP′ was comparable to that of SP′. The EAIs of SP_native_, SP′, and MP′ were 8.25, 9.58, and 9.43 m^2^/g, respectively. Notably, the EAIs of SP-MP′ composites were significantly enhanced, and the values gradually increased as the proportion of MP increased. The EAIs of SP-MP_1:0.5_′, SP-MP_1:1_′, and SP-MP_1:2_′ were 12.53, 15.40, and 21.53 m^2^/g, respectively. A parallel trend was observed for the ESI, with values of 60.9%, 69.1%, 81.6%, 96.4%, 115.7%, and 146.7% for SP_native_, SP′, MP′, SP-MP_1:0.5_′, SP-MP_1:1_′, and SP-MP_1:2_′, respectively. These findings demonstrated that complexation of SP and MP driven by alkaline-thermal treatment enhanced the capacity of protein to form and stabilize oil-in-water emulsions. This improvement was attributed to the alterations in protein characteristics, including solubility, particle size, surface charge, surface hydrophobicity, and conformational change, as discussed previously. Smaller water-dispersible protein particles with a certain surface hydrophobicity facilitated rapid adsorption onto the interfaces of oil droplets, as explained by Shen et al. [34]. Furthermore, protein particles with higher negative charges established a protective coating that enhanced electrostatic repulsion between oil droplets, thereby increasing the resistance of oil droplets to coalescence.

#### 3.9.2. The Particle Size Distributions and Micromorphology of Emulsions Prepared by SP-MP′ Composites

SP-MP′ composites were further utilized to prepare oil-in-water emulsions. As displayed in Figure 6B, all emulsions exhibited uniform monomodal distributions. Compared to E_SPnative_, the distribution curve of E_SP′_ showed a slight shift to left and changes in peak height. The distribution curve of E_MP′_ almost overlapped with that of E_SPnative_. Meanwhile, the distribution curves of E_SP-MP1:0.5′_, E_SP-MP1:1′_, and E_SP-MP1:2′_ were located between E_SP′_ and E_MP′_. There was almost no significant difference in the mean particle diameters D_[3,2]_ among all emulsions, which ranged between 110~120 μm. This may be due to the satisfactory small particle size and large ζ-potential for all proteins, which strongly adsorb onto the oil–water interface and effectively prevent droplet coalescence. As depicted in Figure 6C, optical microscopy images of emulsions revealed that oil droplets for E_SPnative_, E_SP′_, E_MP′_, E_SP-MP1:0.5′_, E_SP-MP1:1′_, and E_SP-MP1:2′_ were homogeneously dispersed, and no significant differences in droplet sizes were observed. This phenomenon differed from the optical microscopy observations of emulsions prepared by pH-shifting treatment of aggregated insoluble soybean protein hydrolysate [31], which reported that the solubility, the flexibility of conformation, and the amount of adsorbed protein on the interface of the protein determined the distribution of emulsion. Overall, emulsion formation was the result of synergistic effects among multiple factors. The SP-MP′ composites possessed the capabilities for stabilizing oil droplets to form uniformly distributed emulsions, which warranted further in-depth investigation into the underlying mechanisms.

## 4. Conclusions

In this study, novel protein composites (SP-MP′ composites) with high solubility were prepared by alkaline-thermal treatment. The original large insoluble protein aggregates were dissociated into smaller ones, and interactions between macadamia protein and soybean protein occurred during the formation of SP-MP′ composites. SP-MP′ composites had better emulsifying abilities and stability, and emulsions prepared by SP-MP′ composites had uniform droplet size distribution. The results of this investigation demonstrated that novel protein composites have a potentiality and prospect as nutritional ingredients, which can conveniently be applied in plant protein beverages. Moreover, further work should explore the interfacial properties of SP-MP′ composites in-depth to interpret the mechanisms of stabilizing the emulsions and developing related products.

## Figures and Tables

**Table 1:** Nitrogen solubility index and secondary structure of SP-MP′ composites *.

Samples	Nitrogen Solubility Index (%)	Maximum Negative Peak Wavelength (nm)	α-Helix	β-Sheet	β-Turn	Random Coil
SPnative	28.6 ± 0.9 e	215.3 ± 0.6 b	10.0 ± 0.6 bc	34.2 ± 0.4 b	22.2 ± 0.4 a	33.6 ± 0.5 a
SP′	90.1 ± 0.3 b	220.3 ± 0.6 a	7.5 ± 0.5 d	36.7 ± 0.5 a	22.3 ± 0.6 a	33.4 ± 0.8 a
MP′	94.0 ± 0.2 a	221.0 ± 1.0 a	14.9 ± 0.6 a	30.1 ± 0.9 c	20.8 ± 0.7 b	34.3 ± 1.0 a
SP-MP1:0.5′	81.1 ± 0.8 d	219.7 ± 0.6 a	11.0 ± 0.8 b	33.7 ± 0.3 b	21.5 ± 0.5 ab	33.8 ± 0.3 a
SP-MP1:1′	82.6 ± 0.2 d	220.3 ± 0.6 a	10.1 ± 0.4 bc	33.9 ± 0.1 b	21.7 ± 0.1 ab	34.2 ± 0.4 a
SP-MP1:2′	84.9 ± 0.9 c	220.0 ± 1.0 a	9.0 ± 0.6 c	34.8 ± 0.9 b	22.3 ± 0.4 a	33.9 ± 0.1 a

* Reported results correspond to mean ± standard deviation. Different letters within the same column indicate significant differences (*p* < 0.05). SP_native_ indicates native soybean protein; SP′ and MP′, respectively, represent soybean protein and macadamia protein after alkaline-thermal treatment; SP-MP_1:0.5_′, SP-MP_1:1_′, and SP-MP_1:2_′ represent SP-MP′ composites obtained by alkaline-thermal treatment at SP/MP mass ratios of 1:0.5, 1:1, and 1:2, respectively.

## Data Availability

The original contributions presented in this study are included in the article. Further inquiries can be directed to the corresponding authors.
